# Detergent/Nanodisc Screening for High-Resolution NMR Studies of an Integral Membrane Protein Containing a Cytoplasmic Domain

**DOI:** 10.1371/journal.pone.0054378

**Published:** 2013-01-22

**Authors:** Christos Tzitzilonis, Cédric Eichmann, Innokentiy Maslennikov, Senyon Choe, Roland Riek

**Affiliations:** 1 Laboratory of Physical Chemistry, Swiss Federal Institute of Technology, ETH-Hönggerberg, Zürich, Switzerland; 2 Structural Biology Laboratory, The Salk Institute, La Jolla, California, United States of America; MRC National Institute for Medical Research, United Kingdom

## Abstract

Because membrane proteins need to be extracted from their natural environment and reconstituted in artificial milieus for the 3D structure determination by X-ray crystallography or NMR, the search for membrane mimetic that conserve the native structure and functional activities remains challenging. We demonstrate here a detergent/nanodisc screening study by NMR of the bacterial α-helical membrane protein YgaP containing a cytoplasmic rhodanese domain. The analysis of 2D [^15^N,^1^H]-TROSY spectra shows that only a careful usage of low amounts of mixed detergents did not perturb the cytoplasmic domain while solubilizing in parallel the transmembrane segments with good spectral quality. In contrast, the incorporation of YgaP into nanodiscs appeared to be straightforward and yielded a surprisingly high quality [^15^N,^1^H]-TROSY spectrum opening an avenue for the structural studies of a helical membrane protein in a bilayer system by solution state NMR.

## Introduction

It is well known that detergent-solubilized membrane proteins for 3D structure determination by X-ray crystallography or NMR spectroscopy often loose in part their function, such as enzymatic activity or ligand binding, along with structural changes [1]–[3]. The nature of this phenomenon is attributed to distortion of an extramembrane domain by detergents or the not well membrane imitation of detergent micelles. For example, a significant surface curvature of the water/micelle interface and less ordered nature of the detergent micelles may result in a deterioration of the 3D structure of both transmembrane and extramembrane domains and increased magnitudes of conformational exchange dynamics and motions [4]. This can lead to the study of a non-relevant protein conformation and non-relevant dynamics [5]. Recently, self-assembling lipid bilayer nanodiscs [6], [7] [5] have been introduced as an alternative approach for reconstitution of integral membrane proteins (IMPs) in a bilayer-mimetic. The nanodisc recreates a native-like lipid bilayer without using detergents. To highlight one of the many membrane mimicking properties of nanodiscs they possess a phase transition from gel to liquid-crystal state similar to that of a pure phospholipid bilayer, though broadened and shifted by a few degrees to higher temperatures attributed to the presence of the membrane scaffold protein (MSP) [6]. Furthermore, the 10–12 nm in diameter large nanodiscs have a thickness of ∼4 nm, which corresponds to the thickness of the biological membrane [4], [6], [8], [9]. In addition, lipid bilayer nanodiscs apparently provide enhanced sample stability when compared with conventional membrane mimetic, which may be a critical property for the long duration of measurements required for the structural and dynamical studies of membrane proteins by solution state NMR [10]–[12]. Furthermore, nanodiscs lack detergents that may destabilize or unfold extra-membranous segments of membrane proteins such as long loops, periplasmic, extra-cellular or cytoplasmic domains, [1] [7] (see also this work below). Finally, reconstitution of membrane proteins in nanodiscs appears to yield a functional entity [10] [13] [4] [14]. The major shortcoming of IMPs reconstituted in nanodiscs for NMR studies is the relatively broad line width of the signals, which are expected to be twice as large as for IMPs embedded in conventional membrane mimetic because of the size of the nanodiscs (∼10 nm). Hence, nanodiscs have been previously used so far mainly as a reference medium to confirm native-like protein fold during detergent screening of membrane proteins for solution NMR studies [14].

In this communication, we describe a NMR-based detergent/nanodisc screening study for high-resolution solution state NMR of the **α**-helical dimeric IMP YgaP from *E. coli*. YgaP has a molecular weight of 18.6 kDa and is composed of 174 residues, of which 119–174 are predicted by the TMHMM [15] to form two transmembrane helices, while residues 1–118 are predicted to form a cytoplasmic rhodanese domain with sulfurtransferase activity [16]–[17]. The function of YgaP is however so far unknown. As we shall see, the analysis of 2D [^15^N,^1^H]-TROSY spectra indicates that detergent solubilization of YgaP perturbed the structural integrity of the cytoplasmic rhodanese domain unless a careful usage of low amounts of mixed detergents was used. In contrast, the incorporation of YgaP into nanodiscs appeared to be straightforward and yielded a surprisingly well [^15^N,^1^H]-TROSY spectrum of full-length protein with properly folded cytoplasmic domain.

## Materials and Methods

### Expression of Full Length YgaP and N-terminal Rhodanese Domain

The pET3a-LIC based plasmid containing the full length YgaP (SwissProt ID P55734) gene was provided by Robert Stroud lab at UCSF [18]. The construction of the N-terminal rhodanese domain plasmid was achieved by inserting, with site directed mutagenesis (Agilent), a stop codon between residues Q109 and P110 of the full-length protein. YgaP C159S mutant (YgaP^−^) was obtained by site-directed mutagenesis (Agilent). YgaP^−^ was expressed, purified and reconstituted in nanodiscs using the same methods, as the ones described below for the wild-type YgaP.YgaP was expressed in *E. coli* strain BL21(DE3) pLysS Star (Invitrogen, Carlsbad, USA). ^2^H, ^15^N labeled YgaP was produced using standard M9 minimal medium [19] based on ^2^H water (Isotec) and 1g/L of ^15^NH_4_Cl (Isotec). Cells were grown at 37°C until OD_600_ = 0.8, and transferred to 18°C for 45 min while shaking. YgaP was expressed by induction with 0.5 mM IPTG (Invitrogen) at 18°C for 12 hours. Expression of the N-terminal rhodanese domain was performed using the same procedure as for the full length YgaP replacing D_2_O by H_2_O.

### Purification of Full Length YgaP

After expression cells were centrifuged at 5,000 g for 10 minutes and resuspended in the lysis buffer (50 mM Tris-HCl pH 8, 300 mM NaCl, 10 mM DTT, 0.5 mg/ml lysozyme (Sigma), protease inhibitor cocktail tablets (Roche)) and incubated at 4°C with gentle stirring for 30 minutes. Cells were lysed by two Microfluidizer (Microfluidics) cycles at 80,000 psi. The lysed cells were centrifuged at 8,000 g for 15 minutes. The supernatant was collected and centrifuged at 100,000 g for 2 hours. The pelleted membrane fraction was resuspended in extraction buffer, 50 mM Tris-HCl pH 8, 300 mM NaCl, 10 mM Imidazole, supplemented with 15 mM DHPC-7 (1,2-diheptanoyl-*sn*-glycerol-3-phosphocholine) plus 1 mM LMPG (1-Myristoyl-2-Hydroxy-*sn*-Glycero-3-Phospho-(1′-rac-Glycerol), 5 mM TCEP (Tris(2-carboxyethyl) phosphine), 10% w/v Glycerol and protease inhibitor cocktail tablets (Roche). YgaP was extracted over night by gentle stirring at 4°C. The solubilized membrane protein fraction was separated from large aggregates by centrifugation at 100,000 g for 45 minutes. The supernatant was loaded on 5 ml Ni Sepharose 6 Fast Flow resin (GE Healthcare), pre-equilibrated with 50 mM Tris-HCl pH 8, 300 mM NaCl, 10 mM Imidazole, 3 mM DHPC-7, 1 mM LMPG, and 1 mM TCEP. The resin was washed with the latter buffer, followed by elution of YgaP with 50 mM Tris-HCl pH 8, 300 mM NaCl, 500 mM Imidazole, 6 mM DHPC-7, 1 mM LMPG, and 5 mM TCEP. Fractions containing the protein were pooled together and the buffer was exchanged using a PD10 desalting column to 20 mM bis-Tris-HCl pH 7, 6 mM DHPC-7, 1 mM LMPG, and 5 mM TCEP. For NMR measurements YgaP was concentrated not more than 10 times [20] using a 30 kDa molecular weight cut-off Centricon (Amicon) concentrator to a final sample concentration of 0.3–0.5 mM. 3% of D_2_O was added to the final sample for the NMR measurements. Size-exclusion chromatography was carried out at a flow rate of 0.5 ml/min on a Superdex 200 10/300GL gel filtration column (GE Healthcare) equilibrated with 20 mM bis-Tris-HCl pH 7, 150 mM NaCl, 3 mM DHPC-7, 1 mM LMPG, and 5 mM TCEP.

The YgaP purification protocol described above yielded the best NMR spectra (see below). For detergent screening the only alteration of the purification protocols were the use of different detergent types and concentrations. In the case of the sample preparation of YgaP in FC12 (Fos-Choline 12) only, the extraction buffer contained 15 mM FC12, while the wash, elution and desalting buffer contained 3 mM FC12. For the sample preparation of YgaP in DHPC-7 only, the extraction buffer contained 15 mM DHPC-7, while the wash, elution and desalting buffer contained 3 mM DHPC-7. For the mixed micelle samples the following condition were used: (A) For the sample preparation of YgaP in DHPC-7/FC12 mixed micelle, the extraction buffer contained 15 mM DHPC-7 and 3 mM FC12, while the wash buffer contained 3 mM DHPC-7 and 3 mM FC12, and the elution and desalting buffer 15 mM DHPC-7 and 3 mM FC12, respectively. (B) For the sample preparation of YgaP in DHPC-7/LMPG, the extraction buffer contained 15 mM DHPC-7 and 1 mM LMPG, the wash buffer 3 mM DHPC-7 and 1 mM LMPG, while the elution and desalting buffer contained 15 mM DHPC-7 and 1 mM LMPG, respectively.

### Purification of the N-terminal Rhodanese Domain of YgaP

The rhodanese domain comprising residues 1–109 of YgaP was purified using the same procedure as described above for YgaP with modifications that include the absence of detergents in the buffers used. In short, after cell lysis and centrifugation the supernatant was loaded on 5 ml Ni^+^ Sepharose 6 Fast Flow resin (GE Healthcare), pre-equilibrated with 50 mM Tris-HCl pH 8, 300 mM NaCl, 10 mM Imidazole, and 1 mM TCEP. The resin was washed with the above buffer and the rhodanese domain was eluted with 50 mM Tris-HCl pH 8, 300 mM NaCl, 500 mM Imidazole, and 5 mM TCEP. Fractions containing the protein were pooled together and the buffer was exchanged using a PD10 desalting column to 20 mM bis-Tris-HCl pH 7, and 5 mM TCEP. For NMR measurements, the rhodanese domain was concentrated using a 10 kDa molecular weight cut-off Centricon (Amicon) concentrator to a concentration of 1–1.5 mM and 3% of D_2_O was added.

### Expression and Purification of MSP1

The plasmid (pET-28a) with the coding sequence of MSP1 fused to a TEV (Tobacco Etch Virus) protease cleavable N-terminal hexahistidine tag was a generous gift of the Arseniev lab. Expression and purification of MSP1 was carried out following the protocol of Shenkarev *et al*., (2009) with modifications. In short, MSP1 was expressed in *E. coli* strain BL21(DE3) Star (Invitrogen, Carlsbad) in Terrific Broth. Cells were grown at 37°C until OD_600_ = 0.8 and protein production was initiated with the addition of 0.3 mM IPTG (Invitrogen, Carslbad). The cultures were incubated initially for 1 hour at 37°C and then the temperature was lowered to 28°C for further 2 hours.

Cells were harvested by centrifugation at 5000 g for 10 min. The cell pellet was resuspended in 100 ml of buffer A (20 mM Tris-HCl, pH 8.0, 0.5 M NaCl) containing 5 mg of DNAse deoxyribonuclease I (Sigma, DN-25), protease inhibitor cocktail tablets and incubated at 4°C with gentle stirring for 20 minutes. 1% Triton X-100 was added to the cell suspension and the solution was incubated with gentle stirring for further 20 minutes at room temperature. The lysate was clarified by centrifugation at 30,000 g for 30 min and applied to a Ni^2+^ resin (25 ml) (Qiagen), which was equilibrated with buffer A containing 1% Triton X-100. The column was sequentially washed with six column volumes of buffer A containing 1% Triton X-100, six volumes of buffer A containing 50 mM of sodium cholate, four volumes of buffer A, and finally eight column volumes of buffer A containing 50 mM imidazole. MSP1 was eluted with buffer A containing 0.5 M imidazole. Purified MSP1 was dialyzed against 10 mM Tris-HCl, pH 7.4, containing 100 mM NaCl and 1 mM EDTA (Ethylenediaminetetraacetic acid). Protein purity was checked by SDS-PAGE.

Proteolytic cleavage of the N-terminal His-tag from MSP1 was performed with the addition of TEV protease for 16 hours at room temperature. The buffer of the reaction mixture was exchanged to 20 mM Tris-HCl, 100 mM NaCl, 50 mM sodium cholate, 10 mM imidazole, pH 8.0 (buffer B) using a PD-10 column (GE Healthcare). The sample was applied to a Ni^2+^ column (volume 15 ml) (Qiagen), which was equilibrated with the same buffer. The flow through containing MSP1^−^ without His tag (MSP1^−^) was collected and dialyzed against 10 mM Tris-HCl, 100 mM NaCl, 1 mM EDTA, pH 7.4. MSP1^−^ was stored for several days at 4°C.

### Reconstitution of YgaP into DMPC Nanodiscs

DMPC (1,2-dimyristoyl-*sn*-glycero-3-phosphocholine) was solubilized in sodium cholate (cholate/DMPC 2∶1 molar ratio) in a glass vial with a teflon-lined screw cap. MSP1^−^ in 10 mM Tris-HCl, 100 mM NaCl, 1 mM EDTA, pH 7.4 buffer was mixed with DMPC cholate solution and incubated for 30 minutes at 27°C while shaking at 150 rpm. YgaP solubilized in detergent micelles was added to the MSP1^−/^DMPC/Cholate mixture and incubated overnight at 27°C while shaking at 150 rpm. Incorporation of YgaP into nanodiscs was initiated with addition of 80% w/v Biobeads SM2 (Biorad) for 2 hours at 27°C while shaking at 150 rpm. The optimal reconstitution of YgaP into nanodiscs was achieved with molar ratio 1∶8∶320∶640, YgaP: MSP1^−^ :DMPC: Cholate. Size-exclusion chromatography of YgaP incorporated in DMPC nanodiscs was carried out at a flow rate of 0.5 ml/min on a Superdex 200 10/300GL gel filtration column (GE Healthcare) equilibrated with 20 mM bis-Tris-HCl pH 7, 150 mM NaCl and 5 mM TCEP. For NMR measurements that required high concentration of protein sample, the solution of YgaP incorporated into nanodics was transferred to a 3 kDa molecular weight cut-off dialysis cassette (Slide-A-Lyzer®, Thermo Scientific, USA) and dialyzed against a solution of 10% Polyethylene glycol (PEG 10K) (Sigma) until the YgaP/nanodisc assembly sample volume was reduced to ∼ 300 µl. YgaP-free nanodiscs were separated from nanodiscs containing N-terminal His-tagged YgaP by Ni^2+^ affinity chromatography.

### NMR Spectroscopy

2D [^15^N,^1^H]-TROSY spectra with a time domain data size of 256*2048 complex points and with t_1max_(^15^N) = 44.8 ms, and t_2max_(^1^H) = 204.9 ms were recorded at 30°C on a Bruker Avance 700 MHz spectrometer equipped with a triple-resonance cryoprobe. The spectra were processed with the program PROSA [21] and analyzed with the program XEASY [22]. The concentration of ^2^H,^15^N- labeled YgaP in NMR samples was ∼80****µM, and ∼300****µM in DMPC and d54-DMPC nanodiscs, respectfully), while the concentration of YgaP in NMR samples with detergents was between 200 and 500 µM.

## Results and Discussion

### Detergent Screening for YgaP

**Figure 1:**
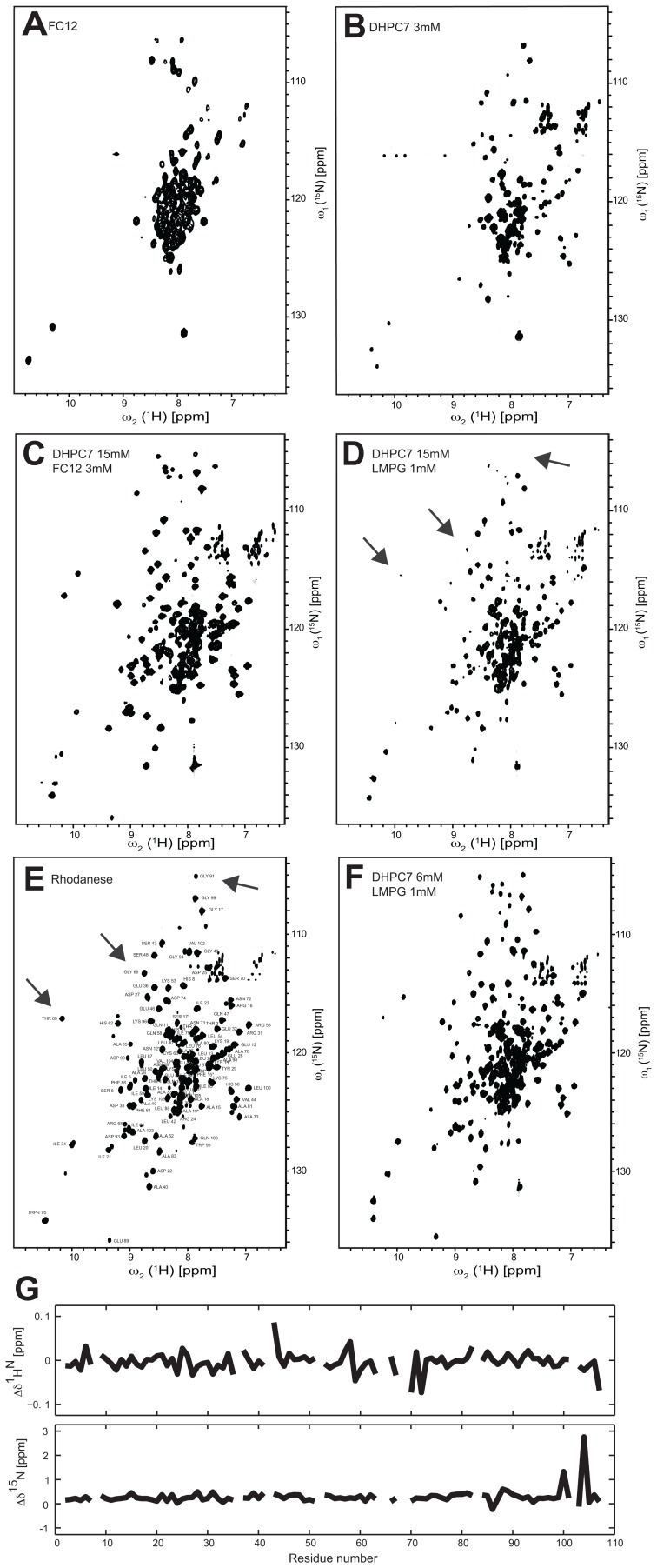
NMR spectra of YgaP in various micellar systems as indicated. 2D [^15^N,^1^H]-TROSY spectra of ^2^H,^15^N-labeled YgaP^−^ in (**A**) FC12, (**B**) DHPC-7, (**C**) DHPC-7 and FC12, (**D, F**) DHPC-7 and LMPG. (**E**) 2D [^15^N,^1^H]-TROSY of the N-terminal rhodanese domain of YgaP. The individual cross peaks are labeled according to the sequential assignment. (**G**) ^1^H and ^15^N chemical shift differences (labeled Δδ^1^H^N^ and Δδ^15^N) between the N-terminal rhodanese domain in solution and the N-terminal rhodanese domain of full length YgaP^−^ in the optimized mixed micellar conditions (i.e. 6 mM DHPC, 1 mM LMPG). The lack of profound up- or down-filed **Δ**δ ^1^H^N^ and **Δ**δ^15^N chemical shift differences indicates the same tertiary structure of the rhodanese domain in solution and in presence of mixed micelles.

**Figure 2:**
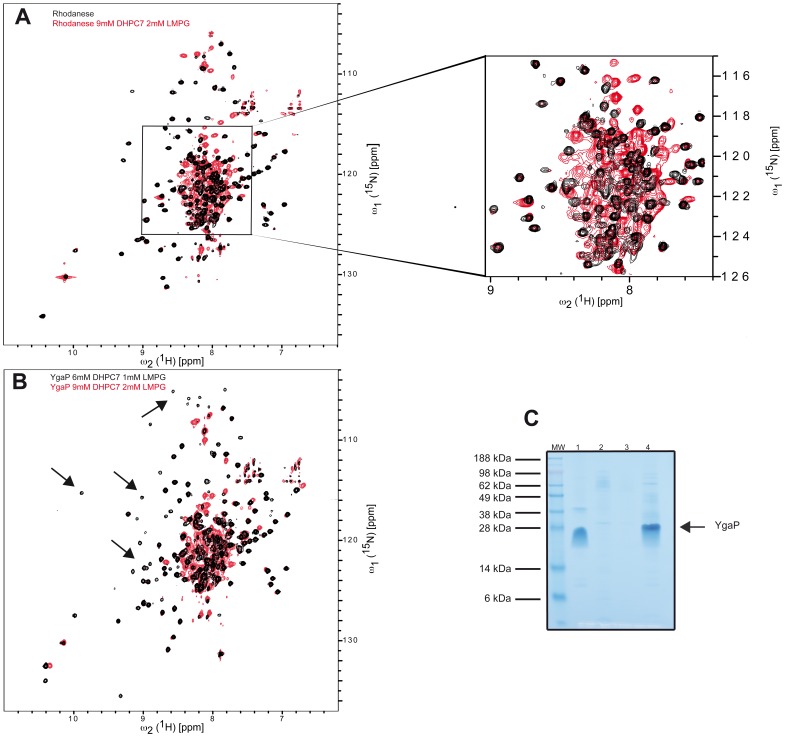
Effects of DHPC-7/LMPG mixed micelles on the N-terminal rhodanese domain and full length YgaP ^−^ **.** (**A**) 2D [^15^N,^1^H]-TROSY spectra of the N-terminal rhodanese domain in absence (Black) and in presence of 9 mM DHPC-7, 2 mM LMPG (Red). For better clarity a portion of the spectrum is magnified as indicated. (**B**) 2D [^15^N,^1^H]-TROSY spectra of ^2^H,^15^N-labeled of YgaP with optimum detergent concentration (i.e. 6 mM DHPC, 1 mM LMPG) (Black) and in presence of 9 mM DHPC-7, and 2 mM LMPG, respectively (Red). Black arrows indicate regions of the red spectrum where resonances are missing, indicating the effect of detergent excess in the quality of the spectrum. (**C**) SDS-PAGE of the nickel affinity purification of YgaP^−^ in DHPC-7/LMPG. 4–12% NuPAGE Bis-Tris gel (Invitrogen, Carslbad). Lanes: (MW) SeeBlue plus2 prestained (Invitrogen, Carslbad), (1) YgaP^−^ after membrane extraction in DHPC-7/LMPG micelles, (2) Loading flow-through fraction of Nickel resin, (3) Washing of Nickel resin, (4) Elution of YgaP^−^ with buffer containing 500 mM imidazole (details of the buffer used are given in the Material and Methods section).

YgaP is an integral *E. coli* membrane protein that has been predicted to have two trans-membrane α -helices using the TMHMM algorithm [15] and an N-terminal cytoplasmic domain. According to Protein Knowledgebase (UniprotKB/Swiss-Prot) the N-terminal domain of YgaP belongs to the rhodanese family of sulfultransferases [23]. YgaP has been previously over-expressed in *E. coli* as well as in a cell free system [18], and has been successfully solubilised by two different detergents n-dodecyl-β-D-maltopyranoside (DDM) and n-Octyl-β-D-glucopyranoside (OG). Based on these findings we developed a protocol for large-scale production of YgaP for solution NMR studies (Figure 1). Expression of YgaP in *E. coli* BL21 cells yielded 15–20 mg of purified protein per liter of M9 isotope enriched minimal media as estimated by absorbance at 280 nm and SDS electrophoresis (Figure 2C). The overexpressed protein was predominantly found in the inner membrane of *E. coli* as tested by cell fractionation of the bacterial membrane, detergent extraction and purification (Figure 2C), suggesting correct targeting and folding. Several detergents (i.e. FC12, DHPC-7 and mixed micelle systems, see Methods) were tested for extraction from the membrane fraction. In all the detergents tested YgaP was extracted from the membrane fraction of *E. coli* with efficiency close to 90%. After a successful purification with Ni affinity chromatography the protein purity was estimated to be higher than 95% (Figure 2C). Since it is our aim to study the structure of YgaP by NMR, we decided to test the homogeneity and integrity of the membrane protein detergent complex by recording 2D [^15^N,^1^H]-TROSY spectra [24] under various detergent conditions and concentrations directly after the affinity purification and buffer exchange without further purification as exemplified in Figure 1. This approach [25] allows us to screen several protein/detergent combinations and correlate the quality of the NMR spectra with the effect that different detergents may have to the conformational state of YgaP. The method is based on the fact that chemical shifts are very sensitive to changes in the chemical environment and therefore can be considered as excellent probes for structural heterogeneity and slow conformational exchange dynamics. Furthermore, using this approach the stability of the protein-detergent complex in relatively high concentrations (∼0.3 mM) and high temperatures (30°C and above), both of which are critical for NMR structure determination, can be evaluated.

Two different zwitterionic detergents with different alkyl chain, FC12 and DHPC-7 were used for recording 2D [^15^N,^1^H]-TROSY spectra of ^15^N, ^2^H- labeled YgaP (Figure 1A–B). Both spectra had significant fewer cross-peaks than expected (i.e. approximately 70 peaks instead of the expected 165) and the peak intensities were not homogeneous. Furthermore, because YgaP has a soluble cytoplasmic rhodanese domain with expected β-sheets and α-helices [17], a broader dispersion of chemical shifts was expected. On top of that YgaP in DHPC-7 aggregated after a few hours at 30°C.

In order to explore the origin of the low sample quality and in particular the lack of the expected chemical shift dispersion, we decided to overexpress and purify the N-terminal cytoplasmic rhodanese domain of YgaP and collect 2D [^15^N,^1^H]-TROSY spectra. It was further rationalized that this spectrum may be used as a reference for the detergent screening towards a wellbehaved YgaP – detergent complex. The 2D [^15^N,^1^H]-TROSY spectrum of ^15^N-labeled N-terminal rhodanese domain (Figure 1 E) shows a chemical shift dispersion expected for a folded protein containing **α**-helixes and **β**-sheets. Subsequent sequential assignment (indicated in Figure 1E) using standard triple resonance experiments [26] on a [^15^N,^13^C]-labeled sample of the N-terminal cytoplasmic domain of YgaP further indicated that it is indeed composed of a rhodanese fold [5] (to be published elsewhere).

Comparing the spectra of the rhodanese domain with those of YgaP in FC12 and DHPC-7 it was assumed that the presence of detergents may perturb the cytoplasmic rhodanese domain of YgaP and that FC12 is even worse doing so than DHPC-7. In contrast YgaP in DHPC-7 was not stable as mentioned above, precipitating within hours. Based on these findings, we decided to screen for a mixture of detergents based on DHPC-7 using little amounts of detergents. The concept of mixed micelles has been used before for solving X-ray structures of membrane proteins [27] and also for solution NMR studies of membrane proteins [25]. Hence, ^15^N,^2^H-labeled YgaP was purified and NMR samples prepared in DHPC-7/FC12 and DHPC-7/LMPG mixed micelles. As shown in Figure 1C and 1D the 2D [^15^N,^1^H]-TROSY spectra have a good chemical shift dispersion of the ^1^H^N^ backbone signals (from 6.8 to 10.4 ppm). Furthermore, most of the cross peaks present in the [^15^N,^1^H]-TROSY spectrum of the rhodanese domain (Figure 1E) have their counterpart in the spectra of the full-length YgaP unlike the spectra acquired of YgaP in FC12 only (Figure 1A). Actually, no significant chemical shift deviations of the sequentially assigned ^15^N-^1^H moieties of the rhodanese domain are observed between the spectra acquired with the rhodanese domain only and full-length YgaP solubilized in mixed detergent micelles (Figure 1G). These findings indicate the structural conservation of the rhodanese domain of YgaP in presence of mixed micelles. In addition, cysteine 159 was replaced by serine in the YgaP construct in order to interfere with potential aggregation through disulfide formation (note, residue 159 is located in the predicted second trans-membrane helix). This mutated construct entitled YgaP^−^ was used for all the subsequent studies.

Although the use of mixed micelles improved significantly the spectra quality of YgaP^−^, a few resonances of the rhodanese domain where still absent (a few examples are indicated by an arrow in Figure 1). Hence, the optimization of the spectra quality was continued by changing the ratio of detergents in mixed micelles and by reducing further the overall amount of detergent in the NMR sample. In parallel, a known amount of both DHPC-7/LMPG detergents (i.e. 9 mM DHPC-7 and 2 mM LMPG) was added to the ^15^N-labeled rhodanese domain accompanied with NMR measurements in order to study directly the effect of detergents on the rhodanese domain (Figure 2A). Indeed, at detergent concentration of 9 mM of DHPC-7 and 2 mM of LMPG, the detergent induced peak losses and/or chemical shift changes in the rhodanese sample (Figure 2A). A similar effect was evident in the [^15^N,^1^H]-TROSY spectrum of full-length YgaP^−^ collected under the same conditions (Figure 2B) although some chemical shift changes and peak losses differ between the two samples. Based on these findings the detergent concentration was lowered further while maintaining the mixed micelle approach yielding an excellent [^15^N,^1^H]-TROSY spectrum at a detergent concentration of 6 mM DHPC-7 and 1 mM LMPG (Figure 1F). In this spectrum cross peaks of the ^15^N-^1^H moieties of the rhodanese domain have their counterpart in the full-length YgaP^−^ mixed micelle complex and no significant chemical shift deviations of the sequentially assigned ^15^N-^1^H moieties of the rhodanese domain are observed between the spectra acquired with the rhodanese domain only and full-length YgaP^−^ solubilized in mixed detergent micelles (Figure 1G). This indicates that the rhodanese domain of the full-length YgaP^−^ in presence of mixed micelles is folded and has the same conformation as that of the free rhodanese domain in solution. In summary, many samples were prepared and many experiments were undertaken to improve the NMR spectra of YgaP, focusing on the inhibition of the unfolding of the cytoplasmic domain of YgaP by the detergents added. This approach yielded finally a high quality [^15^N,^1^H]-TROSY spectrum of YgaP being an excellent starting point for the NMR structure determination of YgaP.

### YgaP^−^ Incorporation into Nanodiscs

Because membrane proteins incorporated in nanodiscs are surrounded by a lipid bilayer, they have been suggested to be a much more attractive membrane mimetic than detergent micelles [1]. This includes structural and dynamical studies by solution state NMR even though nanodiscs are rather large having a diameter of 10–12 nm yielding a long rotational correlation time, which may result in relatively broad peaks in the NMR spectra [13], [14]. In addition to the membrane-alike character of nanodiscs, they lack detergent that may unfold cytoplasmic domains as exemplified above for YgaP. Hence, we decided to test the applicability of nanodiscs as an alternative media for NMR studies of YgaP^−^.

**Figure 3:**
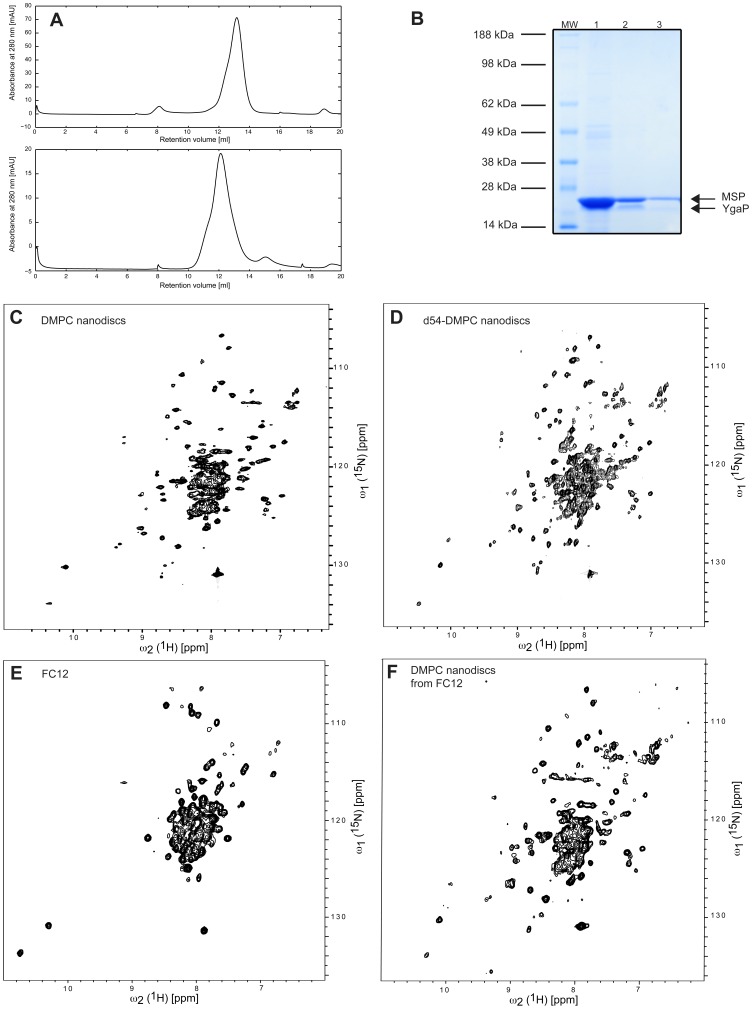
YgaP^−^ incorporation into DMPC nanodiscs. (**A**) Size exclusion chromatography (Superdex 200 10/300GL) of YgaP^−^ in 20 mM bis-Tris-HCl pH 7, 150 mM NaCl, 3 mM DHPC-7, 1 mM LMPG, and 5 mM TCEP. (upper panel) and YgaP^−^ in MSP1/DMPC nanodiscs. (**B**) SDS-PAGE of YgaP^−^ in DMPC nanodiscs. 12% NuPAGE Bis-Tris gel (Invitrogen, Carslbad). Lanes: (MW) SeeBlue plus2 prestained (Invitrogen, Carslbad), (1)-(3) Different dilutions of the YgaP/DMPC nanodisc reaction mixture in the SDS sample buffer after the removal of detergents by Biobeads to resolve the partial overlap due to apparent over-staining in lane 1 for the individual identification of YgaP and MSP1 as indicated. (**C**)–(**D**) 2D [^15^N,^1^H]-TROSY spectra of ^2^H,^15^N-labeled YgaP purified in 6 mM DHPC-7 and 1 mM LMPG and (**C**) incorporated in DMPC nanodiscs or (**D**) in nanodiscs with deuterated d-54 DMPC. (**E**) 2D [^15^N,^1^H]-TROSY spectrum of ^2^H,^15^N-labeled YgaP purified in 3 mM FC12. The sample of (E) was used for a DMPC nanodisc preparation as shown in (**F**): 2D [^15^N,^1^H]-TROSY spectrum of ^2^H,^15^N-labeled YgaP.

The protein was purified as described above using the optimized conditions (i.e. 6 mM DHPC-7 and 1 mM LMPG), and incorporated into nanodiscs, following the published protocol of nanodisc assembly, which is based on the gradual removal of detergents with Biobeads from the sample containing detergent solubilized lipids (i.e DMPC), the membrane scaffold protein MSP and YgaP^−^ in 6 mM DHPC-7 and 1 mM LMPG (see Methods). Several ratios of MSP, DMPC and YgaP^−^ were tested because incorporation of an integral membrane protein in nanodiscs appears to depend mainly on the correct stoichiometry [6]. The incorporation of YgaP^−^ into DMPC nanodiscs was monitored by size-exclusion chromatography (Figure 3A–B), and SDS gel electrophoresis [13], while NMR was used to measure the depletion of the detergents used (i.e 6 mM DHPC-7 and 1 mM LMPG). A ratio of 1∶8∶320 YgaP^−^: MSP: DMPC was found to yield the best NMR spectra (Figure 3C). The cross peaks of the 2D [^15^N,^1^H]-TROSY spectrum (measured for 4 hours) of ∼80 µM YgaP^−^ reconstituted in DMPC nanodiscs are well-dispersed (Fig. 3C). A total of 70 ^15^N-^1^H cross peaks of the rhodanese domain and the C-terminus of YgaP^−^ were unambiguously identified and were found at almost identical positions as in the 2D [^15^N,^1^H]-TROSY spectra of the rhodanese domain only (Figure 1E) with a negligible average change in the chemical shifts. These findings indicate the structural integrity of the cytoplasmic rhodanese domain in YgaP^−^ reconstituted in DMPC nanodiscs.

The [^15^N,^1^H]-TROSY spectrum of YgaP^−^ reconstituted in DMPC nanodiscs shown in Figure 3 was recorded at a protein concentrations of ∼80 <$>\scale 80%\raster="rg1"<$> since attempts to make a more concentrated sample using Amicon concentrators failed. In order to increase the protein concentration to ∼0.3 mM concentration, which is a required concentration if structural and dynamical studies by solution state NMR are attempted, an alternative method of concentration was adopted. The entire assembly reaction after removal of detergents with Biobeads was dialysed against 10% w/v PEG 10K using a dialysis membrane with a 3 kDa molecular weight cut-off. This approach resulted in a several fold reduction of the volume of the YgaP^−/^nanodisc sample and thus an increase in protein concentration yielding an NMR sample of ∼0.3 mM YgaP^−^ assembled in nanodiscs containing deuterated d54-DMPC lipids (Figure 3D). As expected the 2D [^15^N,^1^H]-TROSY of the concentrated sample was similar to the previous diluted sample of YgaP^−^ in DMPC nanodiscs, but has an improved signal to noise. A more detailed analysis reveals the presence of ∼80% of the cross peaks of the rhodanese domain. Furthermore, there are additional ∼40 cross peaks in the [^15^N,^1^H]-TROSY spectrum. If these cross peaks are tentatively assigned to the transmembrane segment of YgaP, also around 80% of the backbone ^15^N,^1^H-moieties of the latter segment show a cross peak in the [^15^N,^1^H]-TROSY spectrum.

In an attempt to further improve the quality of the NMR spectra His-tag YgaP^−^ in DMPC nanodiscs was separated from YgaP^−^ free nanodiscs using metal affinity chromatography (Figure S1). Comparison of the size exclusion chromatography profile (Figure S1B) of the purified YgaP^−/^DMPC nanodisc complex with the one obtained from the unpurified YgaP^−/^DMPC complex (Figure 3B) shows that the complex in both cases have similar molecular weights, and that removal of YgaP^−^ free nanodiscs resulted in a more symmetrical peak shape in the size exclusion profile, indicating a more homogeneous sample. However, the [^15^N,^1^H]-TROSY spectrum does not improve qualitatively upon removal of free nanodiscs (compare Figure S1C with Figure 3D).

While the preparation of YgaP^−^ in nanodiscs yielded straightforwardly a sample with a folded rhodanese domain contrasting the tedious sample preparation optimization in the case of detergents it must be noted that we used already optimized detergent conditions for the extraction of YgaP^−^ from the *E. coli* membrane and purification. In order to test whether the optimized detergent conditions are necessary for the nanodisc preparation of YgaP^−^ we decided to make a sample preparation using non-optimized conditions. YgaP^−^ was purified using 3 mM FC12 yielding a bad quality [^15^N,^1^H]-TROSY spectrum as shown in Figure 3E. The lack of well-resolved cross peaks from the rhodanese domain indicates that FC12 does perturb the structural integrity of the rhodanese domain (Figure 3E). Upon incorporation of this particular sample into DMPC nanodiscs a [^15^N,^1^H]-TROSY spectrum (Figure 3F) is obtained with a significant number of well resolved cross-peaks of the rhodanese domain (note, the difference in the quality of this spectrum with the corresponding one shown in Figure 1E is attributed to the low concentration of ∼50 µM used here). This finding indicates that the rhodanese domain is folded upon YgaP^−^ incorporation into nanodiscs irrespective whether (partial) unfolding happens during the purification procedure caused by the unfolding properties of detergents (Figure 2A).

### Conclusions

The present analysis of the sample preparation of the IMP YgaP indicated that while detergent-micelle preparations require a careful screening of conditions in order to prevent the unfolding of the cytoplasmic rhodanese domain by the detergent used, the incorporation of YgaP into nanodiscs appeared to be straightforward. The obtained high resolution [^15^N,^1^H]-TROSY of YgaP in the optimized mixed micelles on the one hand is considered a promising starting point towards a NMR structure determination. On the other hand the corresponding [^15^N,^1^H]-TROSY spectrum of YgaP incorporated in nanodiscs opens an avenue for solution state NMR towards structural and dynamical studies of a helical integral membrane protein embedded in a lipid bilayer. Both paths are taken in order to exploit the structure and dynamics of YgaP.

## Supporting Information

Figure S1 **Purification of YgaP^−^ incorporated in DMPC nanodiscs.** (**A)** SDS-PAGE of Ni^2+^ affinity purification of YgaP^−^ in DMPC nanodiscs. 12% NuPAGE Bis-Tris gel (Invitrogen, Carslbad). Lanes: (MW) SeeBlue plus2 prestained (Invitrogen, Carslbad), (1) YgaP^−/^DMPC nanodisc reaction mixture before Ni^+^ purification. (2)–(4) Fractions containing MSP1^−^ collected during the loading and washing of the Ni^2+^ affinity column. (5)-(6) Fractions of YgaP^−/^DMPC nanodisc complex eluted from the Ni^2+^ affinity column. (**B)** Size exclusion chromatography (Superdex 200 10/300GL) of Ni^2+^ affinity purified YgaP/DMPC nanodisc complex. (C) 2D [^15^N,^1^H]-TROSY spectra of ^2^H,^15^N-labeled YgaP in DMPC nanodiscs after Ni^2+^ affinity purification of the complex.
(PDF)
