# Supplementary figures and images for: Detergent/Nanodisc Screening for High-Resolution NMR Studies of an Integral Membrane Protein Containing a Cytoplasmic Domain
Source: PLoS One. 2013 Jan 22;8(1):e54378. doi: 10.1371/journal.pone.0054378 (PMC3551814; doi:10.1371/journal.pone.0054378)

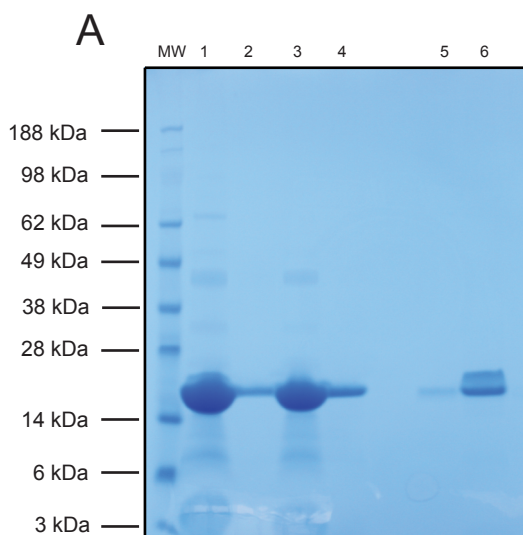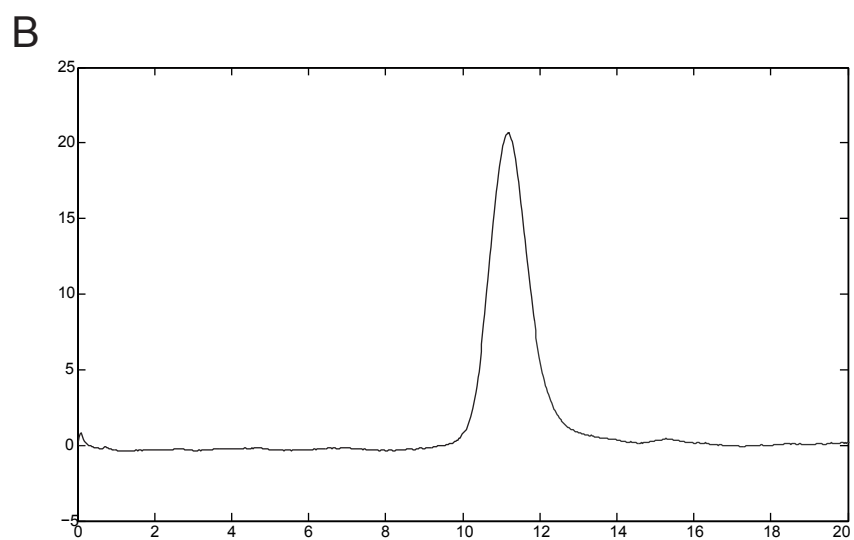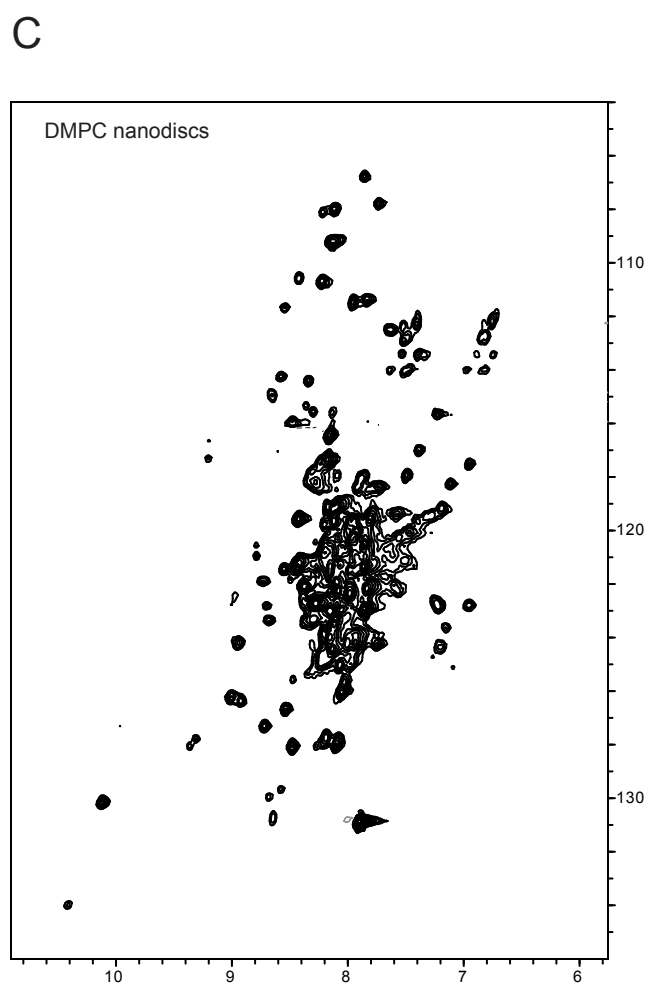

Supplement: Figure S1 — Purification of YgaP− incorporated in DMPC nanodiscs. (A) SDS-PAGE of Ni2+ affinity purification of YgaP− in DMPC nanodiscs. 12% NuPAGE Bis-Tris gel (Invitrogen, Carslbad). Lanes: (MW) SeeBlue plus2 prestained (Invitrogen, Carslbad), (1) YgaP−/DMPC nanodisc reaction mixture before Ni+ purification. (2)–(4) Fractions containing MSP1− collected during the loading and washing of the Ni2+ affinity column. (5)-(6) Fractions of YgaP−/DMPC nanodisc complex eluted from the Ni2+ affinity column. (B) Size exclusion chromatography (Superdex 200 10/300GL) of Ni2+ affinity purified YgaP/DMPC nanodisc complex. (C) 2D [15N,1H]-TROSY spectra of 2H,15N-labeled YgaP in DMPC nanodiscs after Ni2+ affinity purification of the complex. (PDF) [file pone.0054378.s001.pdf]
